# Online Detection of Surface Defects Based on Improved YOLOV3

**DOI:** 10.3390/s22030817

**Published:** 2022-01-21

**Authors:** Xuechun Chen, Jun Lv, Yulun Fang, Shichang Du

**Affiliations:** 1 School of Mechanical Engineering, Shanghai Jiao Tong University, Shanghai 200240, China; cxc1997@sjtu.edu.cn (X.C.); fyl1997@sjtu.edu.cn (Y.F.); 2 Faculty of Economics and Management, East China Normal University, Shanghai 200240, China; jlv@dbm.ecnu.edu.cn

**Keywords:** surface defect detection, YOLOV3, multi-scale detection

## Abstract

Aiming at the problems of low efficiency and poor accuracy in the product surface defect detection. In this paper, an online surface defects detection method based on YOLOV3 is proposed. Firstly, using lightweight network MobileNetV2 to replace the original backbone as the feature extractor to improve network speed. Then, we propose an extended feature pyramid network (EFPN) to extend the detection layer for multi-size object detection and design a novel feature fusing module (FFM) embedded in the extend layer to super-resolve features and capture more regional details. In addition, we add an IoU loss function to solve the mismatch between classification and bounding box regression. The proposed method is used to train and test on the hot rolled steel open dataset NEU-DET, which contains six typical defects of a steel surface, namely rolled-in scale, patches, crazing, pitted surface, inclusion and scratches. The experimental results show that our method achieves a satisfactory balance between performance and consumption and reaches 86.96% mAP with a speed of 80.96 FPS, which is more accurate and faster than many other algorithms and can realize real-time and high-precision inspection of product surface defects.

## 1. Introduction

In the process of industrial production, due to the influence of technological processes, production equipment and site environment, there will be various defects on the product surface. Surface defects not only affect the appearance quality and commercial value of the product itself but also affect the performance of the product and also affect the safety and stability of subsequent deep processing [1]. Therefore, surface defect detection has become a crucial step in industrial production. At present, most detection tasks are completed manually, which has disadvantages of high management difficulty, poor stability, high cost, low efficiency, and low accuracy, and is difficult to meet the demands of automated production of modern enterprises [2].

The defect detection based on machine vision has the advantages of high precision, high efficiency, strong stability, and secondary damage prevention, which provides an optimal scheme for online inspection. Therefore, replacing human eyes with machines has become a trend in industrial surface defect inspection and has been applied in many industrial fields (steel, road, wood, optical components). The existing research on surface defect detection methods can be roughly divided into two categories: a traditional method based on display feature extraction and a deep learning method based on automatic feature extraction. The former is to identify defects by analyzing texture characteristics and extracting features manually, which can be traced back to the 1980s and has rich research achievements. The deep learning method was proposed by Hinton et al. [3], which was successfully applied in the classical image classification task. In the case of sufficient samples, the identification accuracy, robustness and anti-interference ability of deep learning method are far superior to traditional algorithms. Compared with traditional algorithms, the most important advantage is that it weakens the influence of feature engineering on recognition accuracy, adopts supervised and semi-supervised learning to make the network automatically extract the most representative features, simplifies the design difficulty of the algorithm, automatically learns the salient features of the image and completes the task of object detection. For its great performance, many researchers applied the deep learning method to surface defect detection and surpass traditional methods [4,5]. So the deep learning method is widely used in various industrial scenarios and has become the mainstream method of defect detection.

Defect detection can be equated to object detection, and the object is a defect. Compared with classification, object detection can obtain more sufficient defect information, which is convenient for subsequent visual display and quality judgment. Due to the divergent emphasis on detection speed and detection accuracy, the object detection methods are gradually developed into two directions. Faster R-CNN is one of the most representative methods of a two-stage method, which uses a region proposal network (RPN) to generate a candidate box and then achieve classification and position regression. Cheng and Wang [6] applied Faster R-CNN to damage detection of drainage pipes, achieving 83% mAP. Li et al. [7] adopted ZF-Net as the backbone of Faster-RCNN and added a maxpooling layer at the head of the network to adapt defects of large-scale differences, reaching 80.7% mAP. The other is the one-stage method represented by SSD and YOLO, which uses a single-structure network to detect without RPN to generate a candidate box, achieving higher detection speed. Zhang et al. [8] used YOLOV3 with batch re-regularization and focal loss to detect bridge surface damage, which achieved good performance. Yin et al. [9] used YOLOV3 to detect sewage pipeline damage defects and obtained 85.37% mAP. Deng et al. [10] used YOLOV2 with graffiti interference to detect cracks and defects on a concrete surface under complex background. The accuracy of his method was even higher than that of RCNN (mAP 77% vs. 74.5%), and it had higher real-time performance (0.17 s vs. 0.23 s). Thus, the one-stage method is simpler and faster, which is more suitable for end-to-end online defect detection in the industrial field.

Although object detection methods based on deep learning have been partially studied in the industrial field, most of them remain in the laboratory stage and are difficult to be implemented for two reasons. Firstly, surface defects of industrial products are complex and varied in scale, it is hard to detect and locate defects of varying sizes in a wide background area. Secondly, online detection has a very high demand for real-time performance, but most of the research ignores its speed. However, there remains potential for improvement when it is applied to the inspection of surface defects in industrial products.

In this paper, we propose an improved inspection method that is based on YOLOV3 for high-accuracy and high-speed inspection of surface detection. Firstly, we use the MobileNetV2 network as the backbone network in place of the original backbone. Secondly, an enhanced feature pyramid network (EFPN) structure is constructed especially for small-size object detection. At the same time, a new module feature fusing module (FFM) is designed to better integrate the cross-scale features of EFPN. In addition, IoU Loss branches are added to improve the positioning accuracy of the bounding box and narrow the detection gap between the one-stage and two-stage methods. In the end, we evaluate the proposed method on NEU-DET, and the results can demonstrate a clear superiority to other methods.

## 2. Related Work

### 2.1. YOLOV3

Joseph Redmon et al. [10] first proposed the You Only Look Once (YOLO) in 2015. YOLOV3 [11] was developed in 2018 and is one of the state-of-the-art networks. Different from R-CNN series two-stage detection algorithms, YOLO uses a single network structure to complete object tasks. In addition, in YOLOV3, ground truth boxes correspond to positive samples one by one, while in Faster R-CNN, there is a one-to-many relationship, so the number of prediction boxes generated by YOLOV3 is less. These characteristics make YOLOv3 have a higher speed and can reach the level of real-time response, which is more suitable for product surface defect detection tasks in the industrial field.

**Figure 1:**
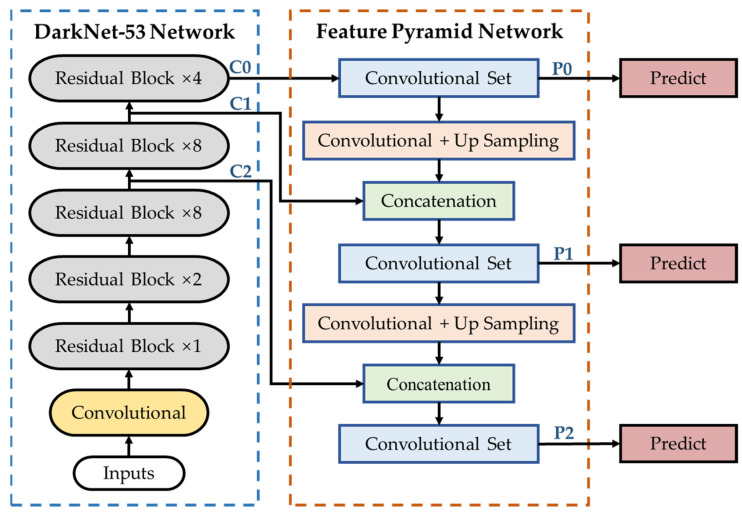
The structure of YOLOV3.

YOLOV3 uses DarkNet-53 as the backbone network, and it is composed of successive residual block which contains 1 × 1 and 3 × 3 convolutional layers and uses shortcut connection, as shown in Figure 1. The feature maps from the backbone are concatenation with the up sampled feature maps. The constructed feature pyramid network outputs three feature maps for bounding box regression and target classification, respectively, to obtain outputs of different scales.

YOLOV3 draws on the residual structure to extract deep feature information and multi-scale feature to improve the performance of different scale objects, especially small objects. However, YOLOV3 trains the complete image, which speeds up the model but weakens the ability to distinguish the target and background. In addition, Darknet-53 is a typical deep network with a huge calculation and a large number of parameters. Using COCO AP as an evaluation indicator, YOLO3 has a weaker performance in accuracy [10]. So YOLOV3 still has some room for improvement in speed and accuracy.

### 2.2. Lightweight Deep Convolution Network

With the popularity of deep learning, convolutional neural network models in the field of computer vision are constantly emerging. From initial 6-layer LeNet to 8-layer AlexNet and from 16-layer VGG16 to 152-layer ResNet, and even developed to DenseNet of thousands of layers [12]. While the performance of the deep learning network has improved, the structure of the network is getting complex, the number of parameters is getting larger and the speed of the network is getting slow, which makes it difficult to perform real-time detection on mobile and embedded devices in the industrial field. Many scholars have made many achievements in network lightweight.

The SqueezeNet [13] used 1 × 1 kernel to replace 3 × 3 kernel to reduce parameter size. In MobileNetV1 [14], depth-wise separable convolution was used to effectively reduce the computational costs. The ShuffleNet [15] used point-wise grouped convolutions and channel shuffle to reduce model computation. These lightweight network models made it possible for mobile devices to run deep learning models. In 2018, the Google team introduced linear bottleneck and inverted residual on the basis of MobileNetV1 and proposed MobileNetV2 [16], which was a widely used lightweight network at present. In this paper, our method is lightened based on this method.

### 2.3. Multi-Scale Features

**Figure 2:**
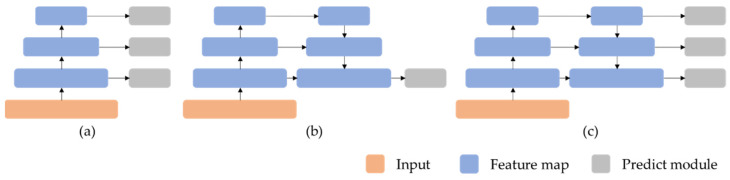
(**a**) Multi-scale feature prediction; (**b**) multi-scale feature fusion + single-scale feature prediction; (**c**) multi-scale feature fusion + multi-scale feature prediction.

The most challenging problem in object detection is object scale variance. In the detection of surface defects, there are great differences within the category of defects, with different shapes and sizes, and even some defects with extremely small, large or extreme shapes (such as slender, narrow and tall, etc.) may appear, which makes it hard to identify and locate defects. This problem can be addressed by using multi-scale features to detect. SSD directly uses different resolution feature maps to detect, as shown in Figure 2a, which results in an independent calculation of each feature’s scale and then slow speed. YOLOV2 fused multi-resolution feature maps into a single map for prediction, and makes shallow information easily be ignored, Figure 2b. FPN [17] adopted multi-scale fusion and prediction, Figure 2c. Followed FPN, evolving MLFPN, NAS-FPN and BiFPN, et al. These FPN variants improved the performance of multi-scale object detection but using the same feature map as the original FPN, which was not enough to deal with tiny defect and is weak for the current small object detection.

## 3. Our Approach

**Figure 3:**
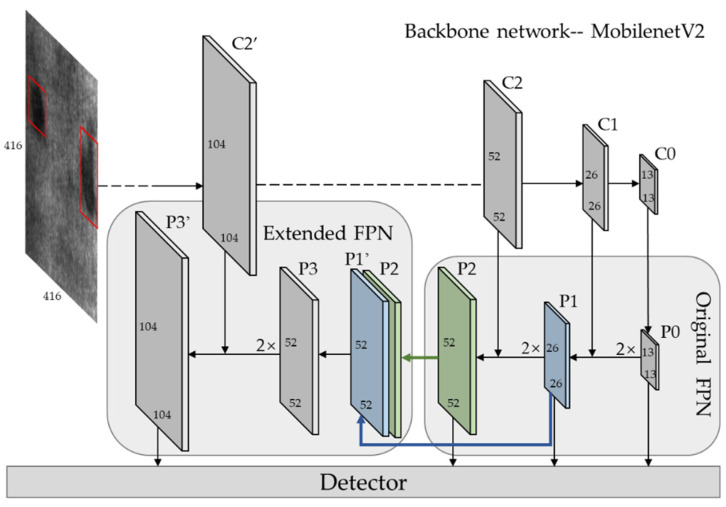
Structure of improved MobileNet-YOLOV3 network. Here Ci denotes the feature map form backbone, and Pi denotes the corresponding feature pyramid layer on FPN/EFPN. The dash line between C2’ and C2 means C2’ and C2 are derived from the same stage and share the same weights. P1, P2 and P3 are origin FPN layers. FFM module integrates P3 from P1 and P2, and then through an top-down framework EFPN to form the extended detection layer P3’. The FPN layers (P0, P1, P2, P3’) will be fed to the following detector to futher detection.

In this section, we introduce our method the improved MobileNet-YOLOv3 in detail, and the network structure is shown in Figure 3. The MobileNetV2 is used as the backbone to replace the original DarkNet-53 with lightweight network architecture. Secondly, an enhanced feature pyramid network (EFPN) is constructed especially for small defect detection, and a feature fusing module (FFM) is designed to integrate the cross-scale features of EFPN. In addition, focal loss and IoU loss functions were used to enhance model accuracy and the learning ability of positive samples.

### 3.1. Feature Extractor

Darknet-53, as the original backbone of YOLOV3, uses successive convolution and pooling layers to obtain a semantic feature map, which greatly improves the detection accuracy, but is weak in speed. MobileNetV2 uses lightweight modules to build a deep structure network, which is widely used in mobile terminals and embedded devices. However, it is not accurate enough for industrial defect detection. Using MobileNetV2 as the backbone, we can get a high-speed and precise network. The improved network structure is shown in Figure 3.

**Figure 4:**
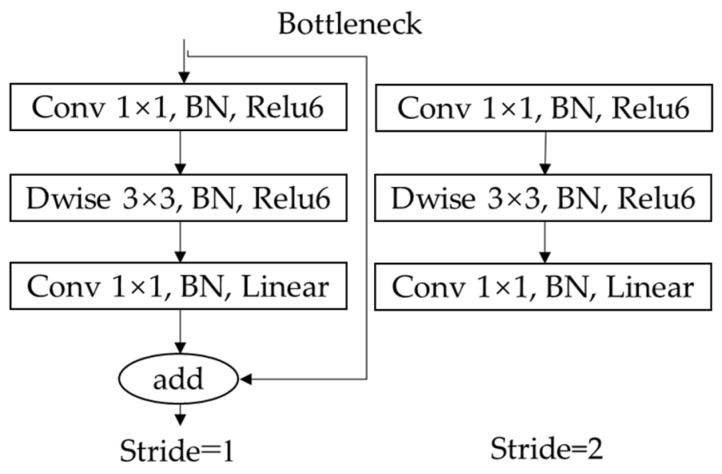
The structure of the Bottleneck module.

Bottleneck is the lightweight core of MobileNetV2, as shown in Figure 4, which has three advantages: Firstly, replace traditional convolution with depth-wise convolution to reduce the number of parameters and computation. Secondly, before convolution, 1*1 expansion layer is added to reduce information loss by increasing input dimension. Shortcut connection is used to construct inverted residuals when stride is 1, so that the network can become deeper and more accurate. In addition, replace activation function at the end of bottleneck with linear to further reduce information loss. Compared with the original backbone DarkNet-53, MobileNetV2 has certain advantages in speed and accuracy.

As a backbone, to compare the performance of Darknet53 and MobileNetV2, see Table 1. The Box AP in the table was obtained by testing the Pascal VOC data set, and the FPS was obtained on Tesla V100. It can be seen that, at nearly the same depth, the MobileNetV2 is only 2/5 the size of DarkNet53. In addition, MobileNetV2 is twice as fast as DarkNet53 with little loss of accuracy, which can meet the real-time requirements of online detection.

**Figure 5:**
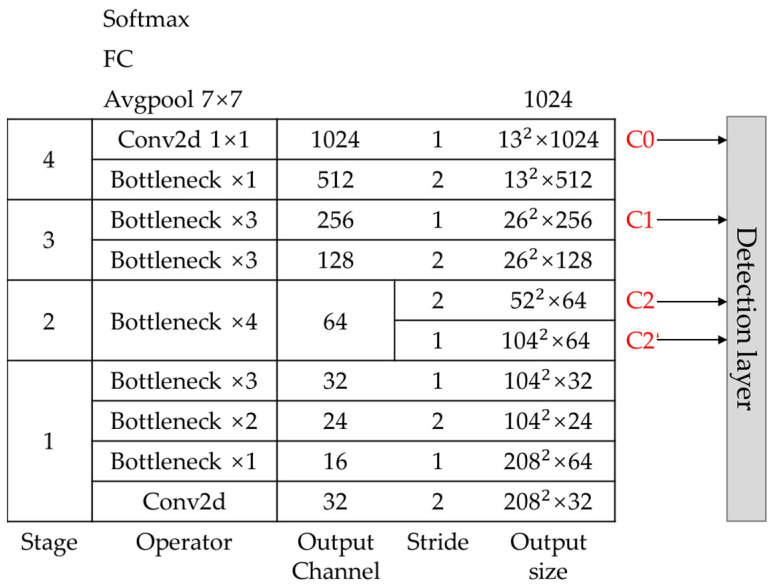
The structure of MobileNetV2 as a feature extractor. Here Ci denotes the feature map from backbone network MobileNetV2. The C2’ and C2 are both derived from the 2nd stage and share the same weights.

In the detection task, avgpool layer, full connected layer and softmax layer after C0 layer in MobileNetV2 are removed, and the structure from input to C0 layer is retained as the backbone network of our method. Input size is [1, 3, 416, 416], and see Figure 5 for detailed parameters of each layer of backbone. The feature map of C0, C1, C2, C2’, which output from stage2, stage3 and stage4, are extracted as the detection layer, with size C0 [1, 1024, 13, 13], C1 [1, 256, 26, 26], C2 [1, 64, 52, 52], C2’ [1, 64, 104, 104], respectively. We remove a convolutional layer of stride 2 in stage2 and get feature map C2’. C2 and C2’ are both outputs of stage2, derived from the same input, and share the same weights, as shown of stage2 in Figure 5.

### 3.2. Extended Feature Pyramid Network

Although the feature pyramid used to fuse three-level feature maps in the detection layer improves the performance of small object detection, the detection of large and medium objects is still coupled together. With the decrease of target size, the performance of the detection will decline rapidly. If the P2 layer is up sampled again following the FPN structure to get a feature map for small-scale detection, the reconstructed image after multiple up sampling will contain a lot of noise and even overwhelm the extraction of semantic information, which will have a negative impact on detection. Therefore, this paper extends a new level, named extended feature pyramid network (EPPN), at the end of the original detection layer, as shown in Figure 3. Firstly, the feature fusing module (FFM) is used to generate a high-resolution feature map P3 on the basis of low-resolution feature map P1. Secondly, P3 is enlarged by up sample and concatenation with C2’ to obtain P3’, which is specially used for small target detection. The feature map reconstructed by FFM not only contains rich semantic information but also has more pixels, which can describe a finer structure and reduce noise interference compared with the up sample.

**Figure 6:**
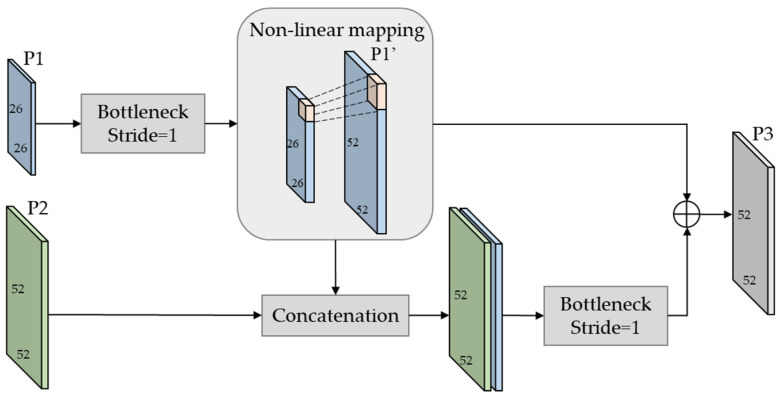
This is the structure of the feature fusing module (FFM). P1 and P2 denotes the feature pyramid layers on FPN. P3 is be obtained with main semantic fearure map P1 and the location reference feature.

Enlightened by the super-resolution method [18], we constructed a feature fusing module (FFM) to reconstruct high-resolution features from low-resolution features with minimum loss, as shown in Figure 6. With feature map P1 as the main input, P2 as the location reference feature, the output P3 can be defined as

(1) $$P3=BN_{c1}\left(P1\right){↑}_{2\times }⊕\;BN_{c2}(BN_{c1}\left(P1\right){↑}_{2\times }||P2)\;\;$$

where$\;BN_{c}\left(\cdot \right)$ denotes bottleneck convolution layer, the function of *c*1 and *c*2 is to extract semantic and location information respectively; ${↑}_{2\times }$ denotes double upscaling by sub-pixel convolution [19]; ⊕ denotes residual connection; || denotes concatenation connection.

Without FFM, the noises in the up sampled P2 will directly affect the extraction of meaningful semantics. However, the feature map P3 obtained by FFM not only contains rich details of small targets which are obtained from upper low-resolution features by super-resolution reconstruction, but also contains location information from lower high-resolution features, and also convolution operation reduces noise interference.

The multi-level output P0, P1, P2 and P3’ are obtained by EFPN, then three anchor boxes are generated in the center of each region. Feature maps in the upper layer use a larger anchor box to capture a large object, and the lower layers use a smaller anchor box to extract small objects. We use K-means clustering to determine our anchor box, and the distance function is defined by IoU so that the error is not affected by box scale, Formula (2) turns into the following:

(2) d(box,centroid)=1−IOU(box,centroid)   

On the NEU-DET dataset, the 12 clusters were in Table 2.

### 3.3. Loss Function

In general, YOLOV3 contains two types of loss functions, category loss (classification) and position loss (regression). Reference RetinaNet [20], the focal loss is adopted for the classification to resolve foreground-background class imbalance as Equation (3) shows. The smooth L1 loss is adopted for the regression to enhance the robustness of loss function to outliers as Equation (4) shows.

As a one-stage detector, YOLOV3 has its natural disadvantage in positioning accuracy compared with Faster RCNN, Cascade RCNN and other two-stage networks. As the classification and positioning branch are independent in the one-stage network, it causes a mismatch between the two branches, so IoU is missing. In order to compensate for the poor performance caused by the low correlation between classification and localization in our module, an IoU prediction head is designed parallel with the regression head at the last layer of regression branch to predict the IoU of prediction box and ground truth box. IoU loss function is established based on IoU-aware single-stage method [21] to predict IoU of the prediction bounding box and ground truth box, modify the score used for NMS, and further improve the prediction performance of YOLOV3. The IoU loss and total loss can be defined as Equations (5) and (6).

(3) $$L_{cls}=\frac{1}{N_{Pos}}\left(\overset{N}{\underset{i\in Pos}{\sum }}FL\left(p_{i},{\hat{p}}_{i}\right)+\overset{M}{\underset{i\in Neg}{\sum }}FL\left(p_{i},{\hat{p}}_{i}\right)\right)\;\;$$

(4) $$L_{loc}=\frac{1}{N_{Pos}}\overset{N}{\underset{i\in Pos}{\sum }}\underset{m\in cx,cy,w,h}{\sum }smooth_{L1}\left(l_{i}^{m}-{\hat{g}}_{i}^{m}\right)\;\;$$

(5) $$L_{IoU}=\frac{1}{N_{Pos}}\overset{N}{\underset{i\in Pos}{\sum }}CE\left(IoU_{i},{\hat{IoU}}_{i}\right)\;\;\;$$

(6) $$L_{total}=L_{cls}+L_{loc}+L_{IoU}$$

where $p_{i}\in \left[0,1\right]$ denotes the predicted probability for category 1, ${\hat{p}}_{i}$ denotes ground truth label, $l_{i}$ denotes the deviation between prediction box and anchor, ${\hat{g}}_{i}$ denotes the deviation between prediction box and ground truth box, $IoU_{i}$ denotes the prediction IoU for each detected box, ${\hat{IoU}}_{i}\;$ represents the target IoU.

## 4. Experiment 

### 4.1. Experience Environment and Evaluation Matric

We used Keras, a deep learning framework, to build our model. The whole experiment was conducted on paddle’s AI studio and is implemented by using Python 3.7. The parameters of the platform were as follows: Tesla V100 GPU, 32 GB Video Memory, 4 Cores CPU, 32 GB RAM, 100 GB Disk. In the experiment, the CUDA 9.0 backend and cuDNN 7.5 were used for GPU acceleration.

Different from image classification, object detection not only needs to predict the correct category of the target but also the location information of the target. In order to evaluate the performance of our object detection task, the following indexes are used:

(1) Precision, Recall, and F1 Score

Precision measures the accuracy of the model prediction, and Recall measures the ability of the model detection for positives. F1-Score is the harmonic mean of Precision and Recall. These indexes are defined as follow:

(7) $$\mathrm{Precision}=\frac{\mathrm{TP}}{\mathrm{TP}+\mathrm{FP}}\;\cdot \;100\%$$

(8) $$\;\;\;\mathrm{Recall}=\frac{\mathrm{TP}}{\mathrm{TP}+\mathrm{FN}}\;\cdot \;100\%$$

(9) F1−Score=2(1Precesion+1Recall)

where TP, FP and FN denote true positive, false positive, and false negative, respectively.

(2) AP and mAP

The mean Average Precision(mAP) is our primary indicator for evaluating model performance. It is the average of Average Precision (AP). AP is calculated by precision and recall whose definition is finding the area under the P-R curve above. AP is usually calculated via the 11-point interpolation average precision calculation method, which can be defined as:

(10) $$\mathrm{AP}=\frac{1}{11}\underset{r\in \left\{0,0.1,\ldots ,1.0\right\}}{\sum }p_{\mathrm{interp}}\left(r\right)\;$$

When we calculate AP for all object classes on all images, we get mAP for all images datasets.

(11) $$\mathrm{mAP}=\frac{\sum_{i=1}^{K}{\mathrm{AP}}_{i}}{K}\;$$

where K denotes the number of categories. When K = 1, mAP = AP.

(3) Params and FPS

Model sizes (Params) are chosen to compare the space complexity of different modules. In addition, we use frames per second (FPS) to show the detection speed.

### 4.2. Datasets and Preprocessing

NEU-DET was used for the experiment, which is a dataset of hot rolled strip steel surface defects released by Northeastern University, and collects six typical defects of steel surface, namely rolled-in scale (RS), patches (Pa), crazing (Cr), pitted surface (Ps), inclusion (In) and scratches (Sc). There were 300 samples for each defect, and a total of 1800 grayscale images, with the original resolution of 200 × 200 pixels. For object detection, the dataset provides bounding box annotations which are saved as an XML document, indicating the category and location of defects in each image.

**Figure 7:**
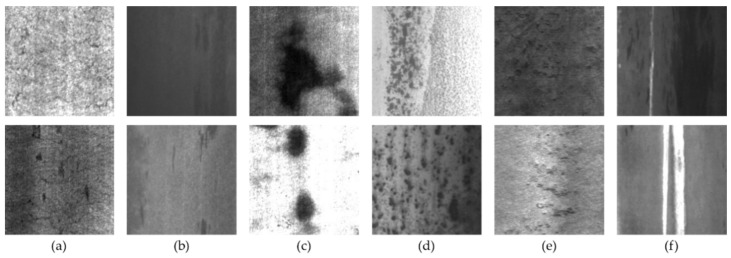
Six kinds of defects in the steel surface. The subset to which the image belongs (**a**) Cr; (**b**) In; (**c**) Pa; (**d**) Ps; (**e**) Rs; (**f**) Sc.

Figure 7 shows part of the data set. Due to the influence of light and the production environment, even the morphologies of similar defects are very different, which puts forward higher requirements for the defect detection model. We use the cross-validation method to train our model, which aims to extract more information from the limited dataset and avoid falling into local minima. So, before the training, we divided the NEU-DET dataset into train set, validation set and test set in a ratio of 7:2:1, containing 1260, 360 and 180 images, respectively. In addition, we use data augmentation operations (mix-up, random distort, random expand, random crop, random horizontal flip) on the train set to improve the diversity of sample data and increase the generalization performance of our model.

### 4.3. Implementation Details

Set the epoch to 300 to start train, and the early-stop strategy was used to terminate the training when the accuracy of validation set decreased or remained flat within 5 epochs. Each epoch contains 79 iterations, in each iteration, the model predicts the category and coordinate of the prediction box. The intersection-over-union (IoU) indicates the ratio of overlapping of predict box and ground truth box. If the IoU of predict box is maximum, we assign a positive label to it, If the IoU is lower than 0.5, we assign a negative label to it, then, the remaining regions are disregarded. Piecewise learning rate decay strategy and momentum optimizer are also used during the training. Initialization parameters of the training process are shown in Table 3.

**Figure 8:**
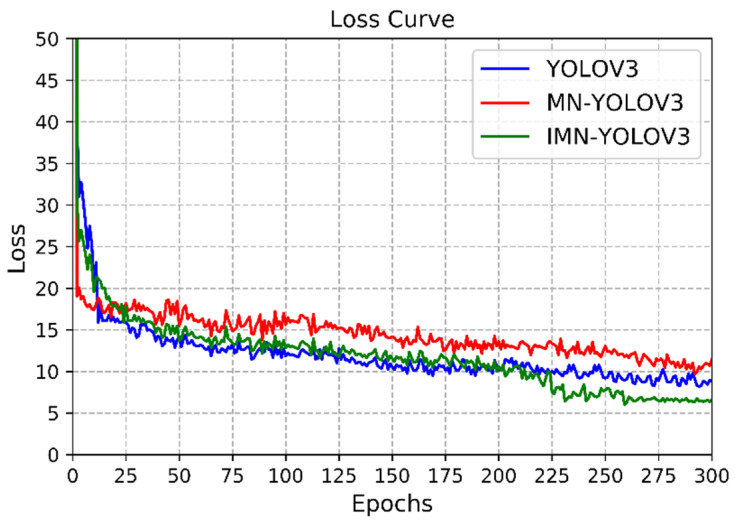
Loss curve of the three YOLO models.

The training process took 5 h to get convergence. Loss represents the gap between the predicted value and the real value, which can be used to evaluate the performance of the model. In addition, the YOLOV3 and MobileNet-YOLOv3 (MN-YOLOv3) were run in the same environment to compare the effects of our model. The training loss curves of the three models are compared as shown in Figure 8.

According to Figure 7, the decreased amplitude of loss is large at the beginning of training, indicating that initial super parameters such as learning rate are reasonable. After a period of training, the change of loss gradually tends to be stable, and not as obvious as that in the initial training period. There is a slight oscillation in the curve, which is related to batch-size setting, but the overall trend is declining. This stage is the fine-tuning stage. When loss hardly changes, the model converges. The YOLOV3, MN-YOLOV3 and improved MN-YOLOV3(IMN-YOLOV3) finally converge to 8.84, 10.58 and 6.45 when the epoch is 285, 280 and 260, respectively. It can be seen that our method has the best performance at final converge loss, which is 2.39 and 4.13 smaller than that of YOLOV3 and MN-YOLOV3. In terms of convergence speed, although MN-YOLOV3 is inferior in the early training stage, our model eventually converges at an earlier epoch.

### 4.4. Experimental Results and Analysis

#### 4.4.1. Experiment Results

**Figure 9:**
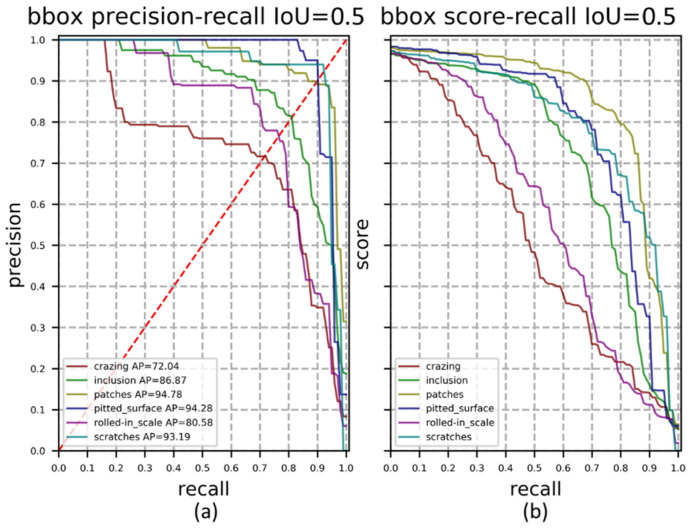
(**a**) P-R curve and BEP; (**b**) score-recall curve. In red dotted line, there have recall equals to precision. The point where the P-R curve intersects the red line is the break-even point.

The test defect images were detected with a trained network and 180 images were completed within 6 s. At the period of testing, all predicted boxes are positive samples, therefore, IoU is set to 0.5 to divide TP, FP and negative samples. The P-R curve of our method for different defect types is shown in Figure 9a. It can be seen from the P-R curve that our method has high detection accuracy and recall rate for In, Pa, Ps and Sc, which have clear boundaries and are quite different from the background. The detection effect on Cr and Rs is not good, these two kinds of defects vary greatly in scale and the boundary is not clear. The P-R curve shows the tradeoff between accuracy and recall at different thresholds and is a useful indicator when there have imbalanced samples. According to the red dotted line in Figure 9a, the break-even point (BEP) with precision equal to recall can be obtained. Figure 9b is the score-recall curve, representing the proportion of objects detected. Compared with the other three defects, Cr and Rs have a smaller curve enclosing area, because there are more defects in one picture, and it is more difficult to detect them completely.

**Figure 10:**
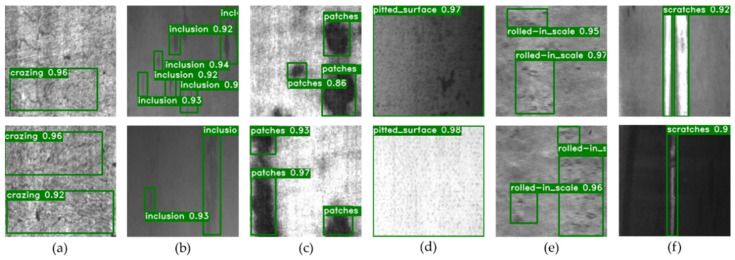
Partial of visual results of detection on NEU-DET. For each image, the green box is the prediction bounding box, and the white label is the class and score. The subset to which the image belongs (**a**) Cr; (**b**) In; (**c**) Pa; (**d**) Ps; (**e**) Rs; (**f**) Sc.

The detection results are shown in Figure 10.

#### 4.4.2. Detection Results Comparison

To verify the performance of the proposed method in the detection of hot rolled strip steel surface defects, we compare it with five other methods which are also for steel defect detection. Table 4 shows the results of detection. In the last three lines of Table 4, it can be seen that the detection accuracy of YOLOV3 varies greatly in different kinds of defect. Ps, Pa and Sc have high detection AP and can be well detected, but the AP of the remaining three kinds of defects is low, especially Cr with AP of 44.70%. The reason is that the prior anchor of YOLOV3 is clustered from the COCO dataset, which cannot well adapt to the detection of NEU-DET which is used in this paper. MN-YOLOV3 uses anchor clustered by NEU-DET and carries out a pre-training operation on the feature extractor. It can be seen that the AP of most defects was significantly improved, especially In, Ps and Rs by 24.13, 17.24 and 17.22 percentage points, respectively. After the improvement of anchor size, the model has stronger adaptability to our dataset, but the AP of Cr and Rs, which have fuzzy boundaries, are still at a low level. When IMN-YOLOV3 is used, the feature map for detection is extended from 3 to 4 layers through EFPN with FFM fusing strategy. The detection accuracy of Cr was significantly improved, which increased from 56.42% to 72.04%, an increase of 15.62%, effectively solving the low detection accuracy of Cr. In addition, AP of other defects is improved also. This result demonstrates that our multilevel features have superior adaptability to different scale defects.

Compared with other methods on NEU-DET detection, it is clear that our method outperforms FRCN, DNN [22], DE_RetinaNet [23], RAF-SSD [24] and ECA+MSMP [25] in terms of mAP. We find that FRCN, DDN [22], DE_RetinaNet [23] and ECA+MSMP [25] are poor for the detection of Cr. Although the AP of RAF-SSD [24] for Cr is 71.10%, which is almost the same as the AP of this method. However, the effect of other defects is not very good, so the mAP is 11.86% smaller than our method. Although RAF-SSD [24] has an AP of 71.10% for Cr, which has reached the same level as our method, the accuracy on other defects is not good, so mAP is 11.86% lower than IMN-YOLOV3. It can be seen that our method can not only effectively detect Cr, the most difficult defect to detect, but also detect other defects more effectively.

#### 4.4.3. Classification Results Comparison

The label corresponding to the IoU with the maximum score and greater than the threshold 0.5 is counted as the category of the image, according to which the whole image classification results of our method can be calculated, namely precision, recall and F1-score indicators. We compared our detection results with traditional classification methods, the results are shown in Table 5. It can be seen that the performance of the proposed IMN-YOLOV3 is better than the other three networks in the classification of various defect categories, especially having a significant improvement in precision, with 14.35%, 3.14% and 4.15% increased.

#### 4.4.4. Real-Time Analysis

Table 6 shows the spatial complexity and the speed of the proposed model and other detection models which has almost the same network depth. As the speed of the model is directly related to the hardware environment, the inference time of the models in the table is obtained by testing validation sets on the Tesla V100 GPU of the Paddle platform with CuDNN 7.5. Including data loading, network forward execution and post-processing, and batch size is 1.

After replacing the backbone of YOLOV3 with MobileNetV2, the Params has been reduced to 2/5 of the original, and the speed is nearly doubled. After extending one layer of the feature map for detection, IMN-YOLOV3 loses a bit of speed but is much more accurate, which is acceptable. Compared with the two-stage method (Faster/Mask-RCNN), the detection speed of our one-stage model can reach more than seven times of theirs, and the model size is also smaller. Under normal circumstances, the maximum speed of steel production is 30 m/s, and the view field of visual equipment is 50–100 cm. To satisfy online detection, the speed of the detection model needs to be between 30–60 FPS. The average speed of our method can reach 81 FPS, which can meet the demand of industrial online detection.

## 5. Conclusions

At present, most surface defects in industrial products were inspected manually, which is time-consuming, too expensive in terms of high labor cost and is prone to misjudgment. How to detect defects online is a bottleneck in industrial production. Aiming at achieving high-precision online inspection, this paper presents an end-to-end detection method based on YOLOV3, called Improved MobileNet-YOLOV3(IMN-YOLOV3). Summarized as follows:

(a) Using MobileNetV2 network instead of VGG16 as the basic network of YOLOV3 algorithm, which makes the model size half and reference time decreased from 20.252 ms to 12.352 ms. Achieving significant improvement in speed.
(b) Proposed EFPN extends the feature map for detection from 3 to 4 layers to obtain more information from different stages. FFM strategy is embedded in the EFPN to efficiently capture features for the extended layer with minimum noise, which significantly improves the detection accuracy, especially the noisiest Cr categories. Indicating that the structure can retain more detailed information while effectively reducing noise.
(c) Use an IoU-aware training loss to solve the mismatch problem between classification confidence and positioning accuracy.

Experiments on the common dataset NEU-DET, IMN-YOLOV3 have obvious advantages in detection accuracy and reference speed. mAP of six categories of strip surface defects reaches 86.96%, and detection speed reaches 81 FPS, realizing the end-to-end high-precision online detection. In addition, sufficient comparative experiments are carried out to demonstrate the performance of our method. In the future, more intelligent data augmentation method, such as GAN, is considered to alleviate the problem of insufficient data in industrial detection to further improve the identification and generalization capabilities of the model.

## Figures and Tables

**Table 1:** DarkNet-53 vs. MobileNetV2.

Backbone	Depth ^1^	Model Size (M)	Box AP ^2^	FPS ^3^
DarkNet-53	50	249.2	31.0	54.977
MobileNetV2	52	100.7	29.9	104.291

^1^ Depth, the network depth of the remaining part of the network when the top classifier is removed; ^2^ Box AP, box average precision; ^3^ FPS, frames per second.

**Table 2:** Parameters of three anchor boxes.

Feature Map Form	Feature Map Dimensions	Scale of Anchor Box [w, h]	Total Number of Anchor Box on This Feature Map
P0 ^1^	13 × 13	[128, 351]; [262, 228]; [325, 392]	13 × 13 × 3
P1	26 × 26	[55, 318]; [156, 169]; [301, 130]	26 × 26 × 3
P2	52 × 52	[45, 101]; [97, 121]; [291, 56]	52 × 52 × 3
P3′	104 × 104	[36, 80]; [55, 163]; [107, 90]	104 × 104 × 3

^1^ P0, the corresponding feature map in Figure 3.

**Table 3:** Initialization parameters of our method.

Parameters	Value	Note
Size of input images	416 × 416	data
Loss function	$L_{total}$	$L_{cls}+L_{loc}+L_{IoU}$
Optimizer	Momentum	0.9
Batch size	16	
Training epochs	300	
Learning rate (lr)	0.000125	
lr_decay_epochs	[216, 240]	The epoch where the lr declines
lr_decay_gamma	0.1	lr decay rate

**Table 4:** Detection results on NEU-DET.

Method	Backbone	mAP	Cr	In	Pa	Ps	Rs	Sc
FRCN	ResNet50	77.9	52.5	76.5	89.0	84.7	74.4	90.3
DDN [22]	ResNet50	82.3	62.4	84.7	90.7	89.7	76.3	90.1
DE_RetinaNet [23]	ResNet50	78.25	55.78	81.91	94.69	89.24	70.17	77.70
RAF-SSD [24]	ResNet50	75.10	71.10	75.50	80.10	72.60	75.30	75.40
ECA+MSMP [25]	ResNet50	80.86	55.61	77.84	93.90	74.43	89.72	93.66
YOLOV3	DarkNet53	69.10	44.70	60.80	84.40	74.50	61.10	87.20
MN-YOLOV3 ^1^	MobileNetV2	82.90	56.42	84.93	93.78	91.74	78.32	92.19
IMN-YOLOV3 ^2^	MobileNetV2	86.96	72.04	86.87	94.78	94.28	80.58	93.19

^1^ MN-YOLOV3, YOLOV3 with improved anchor size and pretrained operation; ^2^ IMN_YOLOV3, MN-YOLOV3 with EFPN structure and FFM fusing module.

**Table 5:** Classification results on NEU-DET.

Method	Task	Precision	Recall	F1-Score
VGG16+CBAM ^1^ [26]	Classification	84.02	81.03	82.50
ResNet50+CBAM	Classification	95.23	95.15	95.19
MobileNetV2+CBAM	Classification	94.22	95.33	94.77
IMN-YOLOV3	Classification+location	98.37	95.48	96.90

^1^ CBAM: the attention mechanism is to improve the performance of classification in NEU-DET.

**Table 6:** Space complexity and detection speed of these models on NET-DET.

Method	Backbone	Params (M)	Inference Time (ms/Image)	FPS
Faster RCNN	ResNet50	136.0	78.450	12.747
Mask R-CNN	ResNet50	143.9	86.096	11.615
SSD	VGG16	140.5	21.736	46.007
YOLOV3	DarkNet53	249.2	20.252	49.377
MN-YOLOV3	MobileNetV2	99.2	11.834	84.502
IMN-YOLOV3	MobileNetV2	107.4	12.352	80.959

## Data Availability

The publicly archived hot rolled strip steel datasets NEU-DET can be download in the folloing link: http://faculty.neu.edu.cn/songkechen/zh_CN/zhym/263269/list/index.htm (accessed on 12 December 2021).
